# The roles of factor Va and protein S in formation of the activated protein C/protein S/factor Va inactivation complex

**DOI:** 10.1111/jth.14594

**Published:** 2019-08-09

**Authors:** Magdalena Gierula, Isabelle I. Salles‐Crawley, Salvatore Santamaria, Adrienn Teraz‐Orosz, James T. B. Crawley, David A. Lane, Josefin Ahnström

**Affiliations:** ^1^ Centre for Haematology Imperial College London London UK

**Keywords:** activated protein C, factor Va, phopsholipids, protein S, prothrombinase

## Abstract

**Background:**

Activated protein C (APC)‐mediated inactivation of factor (F)Va is greatly enhanced by protein S. For inactivation to occur, a trimolecular complex among FVa, APC, and protein S must form on the phospholipid membrane. However, direct demonstration of complex formation has proven elusive.

**Objectives:**

To elucidate the nature of the phospholipid‐dependent interactions among APC, protein S, and FVa.

**Methods:**

We evaluated binding of active site blocked APC to phospholipid‐coated magnetic beads in the presence and absence of protein S and/or FVa. The importance of protein S and FV residues were evaluated functionally.

**Results:**

Activated protein C alone bound weakly to phospholipids. Protein S mildly enhanced APC binding to phospholipid surfaces, whereas FVa did not. However, FVa together with protein S enhanced APC binding (>14‐fold), demonstrating formation of an APC/protein S/FVa complex. C4b binding protein‐bound protein S failed to enhance APC binding, agreeing with its reduced APC cofactor function. Protein S variants (E36A and D95A) with reduced APC cofactor function exhibited essentially normal augmentation of APC binding to phospholipids, but diminished APC/protein S/FVa complex formation, suggesting involvement in interactions dependent upon FVa. Similarly, FVa^Nara^ (W1920R), an APC‐resistant FV variant, also did not efficiently incorporate into the trimolecular complex as efficiently as wild‐type FVa. FVa inactivation assays suggested that the mutation impairs its affinity for phospholipid membranes and with protein S within the complex.

**Conclusions:**

FVa plays a central role in the formation of its inactivation complex. Furthermore, membrane proximal interactions among FVa, APC, and protein S are essential for its cofactor function.

**Essentials**

- The APC/protein S/FVa complex needed for FVa inactivation has proven elusive to investigate.
- Complex formation on phospholipid membranes was investigated using binding and functional assays.
- FVa and protein S synergistically enhance association of APC to negatively charged phospholipids.
- Protein S Gla36, Asp95, and FV Trp1920 are required for formation of the FVa inactivation complex.

## 1 INTRODUCTION

Factor V (FV) is an important regulator of blood coagulation. Its activated form, FVa, functions as a cofactor for activated factor X (FXa), increasing the rate of thrombin generation by four to five orders of magnitude.^1^ FVa is, in turn, regulated by activated protein C (APC). In this inhibitory pathway, FVa is proteolysed by APC, leading to the loss of its FXa cofactor function.

Factor V is a large 330 kDa single‐chain plasma protein (20‐30 nmol/L).^2^, ^3^, ^4^ It is comprised of three A domains, a highly glycosylated B domain, and two C domains arranged A1‐A2‐B‐A3‐C1‐C2. FV is converted to FVa by thrombin or FXa, leading to the release of the large B domain and exposure of the FXa‐binding sites.^3^, ^5^, ^6^ FVa is proteolytically inactivated by APC at three sites, Arg306, Arg506, and Arg679, all located within the A2 domain.^3^

The importance of FV for normal haemostasis is highlighted by the bleeding diathesis associated with FV deficiency,^7^, ^8^, ^9^ as well as by the increased risk of deep vein thrombosis (DVT) caused by partial resistance to APC‐mediated proteolysis.^10^ Among the most common risk factors for DVT is the FV^Leiden^ mutation (R506Q), which leads to diminished inactivation by APC.^11^ Another FV mutation, W1920R, termed FV^Nara^, was recently associated with greater APC resistance than FV^Leiden^.^12^ Unlike other FV mutations that cause APC resistance,^13^, ^14^ the W1920R substitution is located in the C1 domain, which is spatially separated from the APC cleavage sites in the A2 domain. Consequently, the mechanism of APC resistance by the W1920R mutation is unclear, but it was hypothesized to disrupt the FVa/APC interaction.^12^

The inactivation of FVa by APC is highly dependent on their binding to phospholipid surfaces and is further augmented by protein S. Whereas cleavage at Arg506 by APC is only mildly stimulated by protein S, cleavage at Arg306 is enhanced by 20‐ to 30‐fold.^15^, ^16^ Protein S circulates either free or bound to C4b‐binding protein (C4BP).^17^ It is the free form of protein S that most effectively functions as a cofactor for APC, suggesting that the binding of C4BP to protein S may block important functional interaction sites.^17^, ^18^, ^19^ Numerous studies have attempted to identify residues involved in the APC/PS/FVa interactions. Gla36 and Asp95 within the protein S Gla and EGF1 domains, respectively, are both essential for the APC cofactor function of protein S.^20^, ^21^ Similarly, Asp36, Leu38, and Ala39 in protein C are essential for protein S‐dependent enhancement of APC function.^22^ However, despite their importance, the precise mechanistic role that these residues fulfil is unknown.

Different molecular mechanisms have been proposed to describe how protein S enhances APC function, including protein S causing a conformational change in APC upon interaction. Such a conformational change could potentially explain the preferential enhancement of APC‐mediated cleavage of FVa Arg306 by protein S.^23^ It has also been suggested that protein S exerts is cofactor function through ~10‐fold enhancement of APC binding to negatively charged phospholipid membranes.^24^ However, it is unlikely that this is the only cofactor mechanism as this alone would fail to explain the differential enhancement of cleavages of the Arg306 and Arg506 in FVa. FVa has also been reported to increase the affinity of APC to phospholipids, but to what extent and how this is influenced by protein S is unclear.^25^ In the present study, we aimed to elucidate the nature of the phospholipid‐dependent interactions among APC, protein S, and FVa.

## 2 METHODS

### 2.1 Protein S, FV, and C4BP

Protein S and protein S variants were expressed in stably transfected HEK293 cells (ATCC) followed by a two‐step purification using barium citrate precipitation and anion exchange chromatography as previously described (Figure S1A).^20^ Following purification, protein S was quantitated using A280 with extinction coefficient (E1%, 1 cm) of 9.8 as previously described.^26^

FV^Nara^ (W1920R) mutation was introduced by site‐directed mutagenesis into a vector (pED) containing wild‐type (WT) FV vector (kindly supplied by Dr Rodney Camire, University of Pennsylvania). Full‐length WT FV and FV^Nara^ were expressed and purified as described previously (Figure S1B).^6^, ^27^ The concentration of WT FV was determined functionally using prothrombinase assays^21^ and by absorbance at 280 nm.^6^, ^27^ The concentration of FV^Nara^ was determined using absorbance at 280 nm and confirmed using semi‐quantitative Western blotting using WT FV as a standard. For experiments involving FVa, FV was activated by human α‐thrombin (Enzyme Research Laboratories), followed by addition of hirudin, as previously described.^21^ Full activation was confirmed by Western blotting (data not shown).

β‐chain‐containing C4BP (and protein S‐free) was purified from pooled fresh frozen citrated human plasma as previously described.^28^, ^29^, ^30^

### 2.2 Phospholipid vesicle preparation

Phospholipids (Avanti Polar Lipids) 1,2‐Dioleoyl‐sn‐glycero‐3‐phosphocholine (DOPC), 1,2‐Dioleoyl‐sn‐glycero‐3‐phosphoserine (DOPS), and 1,2‐Dioleoyl‐sn‐glycero‐3‐phosphoethanolamine (DOPE), 1,2‐Dioleoyl‐sn‐glycero‐3‐phosphoethanolamine‐N‐biotinyl (biotinylated DOPE) were mixed and extruded as described previously.^21^

### 2.3 Preparation of phospholipid coated magnetic beads

Phospholipid coated magnetic beads were prepared as previously described with minor modifications.^31^ Streptavidin coated beads (Invitrogen) were washed twice with 0.5% BSA in 20 mmol/L Tris, pH 7.4, 150 mmol/L NaCl (TBS), 5 mmol/L CaCl_2_ and quenched with 2% BSA in TBS, 5 mmol/L CaCl_2_ for 4 hours at 37°C with mixing. Subsequently, the beads were mixed with 2 mmol/L phospholipid vesicles (DOPC/DOPS/DOPE/biotinylated DOPE; 60:20:18:2) at room temperature, overnight with rotation. Finally, the beads were washed twice and re‐suspended in 0.5% BSA in TBS, 5 mmol/L CaCl_2_ at 2.5 mg/mL.

### 2.4 Pull‐down assays using phospholipid‐coated beads

To assess the effect of protein S and/or FVa on APC binding to phospholipids, phospholipid‐coated magnetic beads (250 μg/mL) were pre‐incubated with 100 nmol/L protein S and/or 25 nmol/L FVa for 25 minutes prior to addition of 50 nmol/L fluorescently labelled/active‐site blocked APC, APC‐Fluor (APC‐DEGR), which has previously been used successfully to study APC functions^25^, ^32^, ^33^; Haematologic Technologies Inc. (HTI). Supernatants and beads were separated with a magnet after 2 minutes, and the beads washed briefly with TBS containing 5 mmol/L CaCl_2_. Bound proteins were eluted with LDS buffer (Invitrogen). Samples were analyzed by Western blotting using polyclonal anti‐protein C (Sigma). Subsequently the membranes were stripped and re‐probed using monoclonal anti‐FV (AHV‐5146; HTI) and polyclonal anti‐protein S (Dako) antibodies.

WT FVa and FVa^Nara^ binding to phospholipids was assessed by incubating phospholipid‐coated magnetic beads with 5‐25 nmol/L FVa/Va^Nara^ in 100 μL. After 25 minutes, supernatants were collected and beads washed with TBS containing 5 mmol/L CaCl_2_. Bound proteins were eluted with 30 μL LDS buffer and 15 μL of each fraction (supernatant and eluted sample) were analyzed by Western blotting with anti‐FV (AHV‐5146) antibodies.

### 2.5 APC binding to phospholipid‐coated beads by flow cytometry

Phospholipid‐coated magnetic beads (125 μg/mL) were pre‐incubated with 100 nmol/L protein S and/or 0‐75 nmol/L FVa at room temperature for 25 minutes after which 0‐75 nmol/L APC‐Fluor was added. The geometric mean fluorescence (mean fluorescence intensity; MFI) was measured over time by flow cytometry (BD FACSCalibur). Data were analyzed using Flowlogic software. To examine the influence of C4BP‐binding to protein S upon enhancement of APC binding to membranes, 200 nmol/L ß‐chain‐containing C4BP was pre‐incubated with protein S and FVa for 10 minutes, prior to the addition of APC‐Fluor. All flow cytometry data (histograms excluded) are presented as change in geometric means fluorescence intensity (ΔMFI) where the auto florescence from the phospholipid coated magnetic beads has been subtracted.

### 2.6 Thrombin generation assay

Thrombin generation assays were performed using calibrated automated thrombography (CAT) to assess APC cofactor function of protein S variants as described previously.^20^, ^21^ Thrombin generation was initiated in protein S‐depleted plasma (Enzyme Research Laboratories) with 1 pmol/L tissue factor (Dade Innovin) in the presence of 50 μmol/L phospholipids (DOPC/DOPS/DOPE; 60:20:20), in the presence and absence of 9 nmol/L APC (HTI) and 0‐120 nmol/L protein S.

### 2.7 Prothrombinase assays

Prothrombinase assays were performed essentially as described previously.^12^, ^20^, ^34^ Prothrombin activation was initiated by addition of 0‐600 nmol/L prothrombin to a mixture of 8pM FVa, 5 nmol/L FXa, in the presence of 0‐50 μmol/L phospholipids (DOPC/DOPS; 90:10) and 2 mmol/L CaCl_2_ in 25 mmol/L Tris (pH 7.4), 150 mmol/L NaCl containing 0.5 mg/mL ovalbumin.^34^

To quantify the remaining FVa activity after APC‐mediated inactivation, assays were performed using 500 nmol/L prothrombin. Due to the reduced cofactor function of FVa^Nara^, 16 pmol/L was used.

Reactions were allowed to proceed for 2 minutes at 37°C and then stopped by dilution into TBS, 20 mmol/L EDTA, 1% PEG 6000. Prothrombin activation was quantified by cleavage of S‐2238 (Chromogenix) and comparison to a thrombin standard curve.^21^

### 2.8 FVa inactivation assay

FVa inactivation assays were used to monitor the inactivation of FVa and FVa^Nara^ by APC, as well as the ability of protein S and protein S variants to enhance APC‐mediated FVa degradation.^20^, ^21^ Briefly, FVa was incubated with 0‐5 nmol/L APC in the presence of 0‐100 nmol/L protein S and 25 μmol/L phospholipids (DOPC/DOPS/DOPE; 60:20:20) in 25 mmol/L Tris (pH 7.4), 150 mmol/L NaCl, 5 mmol/L CaCl_2_ containing 5 mg/mL BSA. To specifically characterize the enhancement of cleavage at FVa Arg306 mediated by protein S and its (E36A) and (D95A) variants, FV R506Q/R679Q was used.^16^ The inactivation reaction was allowed to proceed for 10 minutes at 37°C. Thereafter, the remaining FVa activity was measured using prothrombinase assays.

## 3 RESULTS

### 3.1 Influence of protein S and FVa upon APC association with phospholipid surfaces

**Figure 1:**
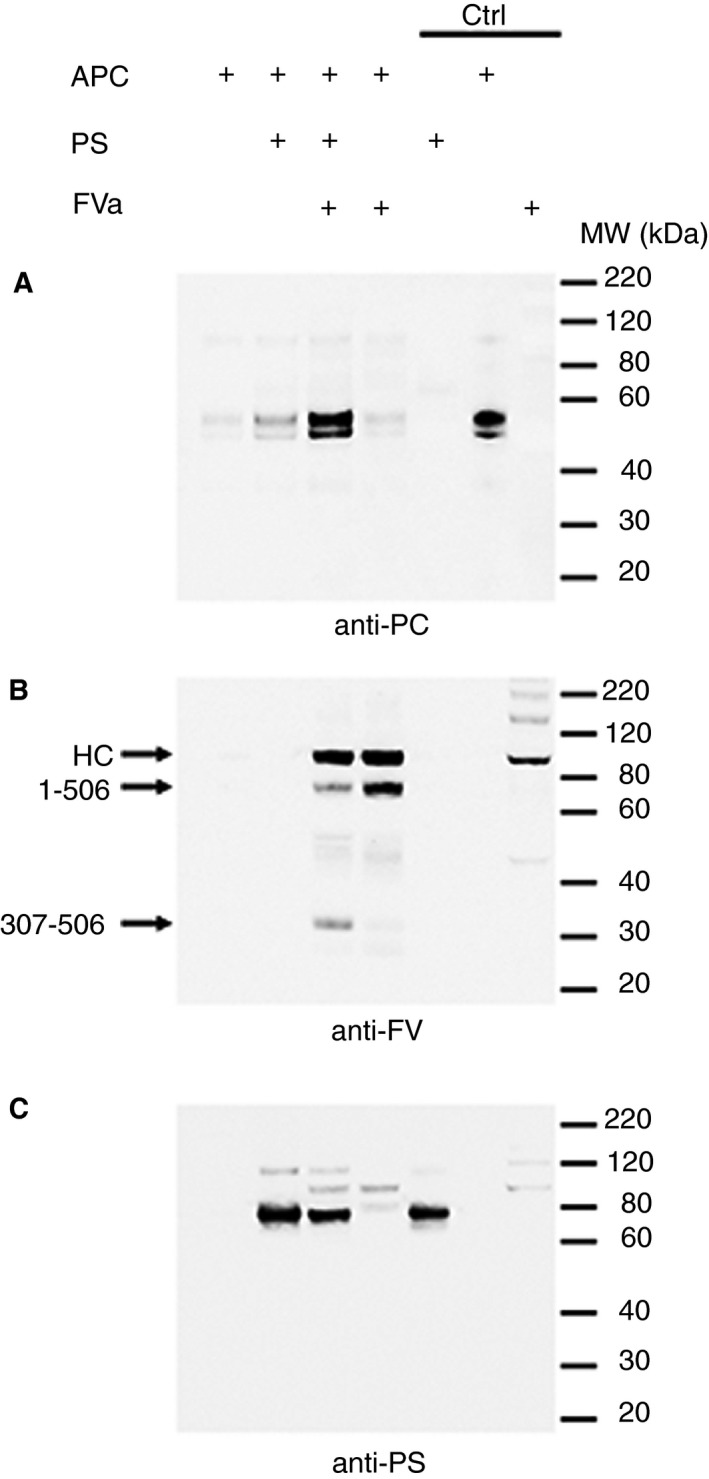
Enhancement of APC binding to phospholipids by protein S and FVa. Binding of 50 nmol/L APC‐Fluor to phospholipid coated magnetic beads was analyzed in pull‐down experiments. APC binding was assessed in the presence and absence of 100 nmol/L protein S and/or 25 nmol/L FVa. Phospholipid‐bound proteins were pulled down using a magnet and eluted after 2 min incubation. Bound proteins were separated by SDS‐PAGE (4%‐12% gradient gel), transferred to nitrocellulose membranes and bound APC was detected using rabbit polyclonal anti‐protein C antibodies (A). Subsequently the membranes were stripped and re‐probed using anti‐FV and anti‐protein S antibodies for FVa (B) and protein S (C) detection, respectively. Bands detected in the ~90 to 120 kDa range of the protein S blot (C) are residual from the detection of FVa. The three lanes labelled controls (Ctrl) are positive controls where pure proteins have been loaded as indicated. Representative blots are shown (n = 3). HC, FVa heavy chain. 1‐506 and 307‐506 points out FVa cleavage products resulting from cleavage at Arg506 only or at Arg306 and Arg506, respectively

Protein S has been suggested to enhance APC binding to negatively charged phospholipid membranes.^24^ To test this hypothesis, and also to study how FVa influences APC binding to phospholipids, pull‐down experiments using active‐site inhibited APC (APC‐Fluor) in the presence of protein S and/or FVa were performed. After 2 minutes incubation of APC‐Fluor with phospholipid‐coated magnetic beads in the presence and absence of 100 nmol/L protein S and/or 25 nmol/L FVa, bound proteins were analyzed by Western blotting. APC‐Fluor alone bound to the beads with low efficiency (Figure 1A). The presence of FVa had no discernible effect on the amount of APC bound to the beads. While the APC binding was moderately increased by the addition of protein S, APC association was appreciably augmented in the presence of both protein S and FVa (Figure 1A). This suggests that protein S and FVa synergistically enhance the association of APC to phospholipids. Re‐probed blots showed limited signs of FVa proteolysis (Figure 1B), consistent with the ~0.2% APC residual activity found in the APC‐Fluor. Protein S binding was largely unaffected by addition of FVa to APC (Figure 1C). These results demonstrate that active‐site blocked APC‐Fluor enables stable complex formation and can be used to study this mechanism in more detail.

### 3.2 Quantitation of the FVa and protein S‐mediated enhancement of APC phospholipid binding

**Figure 2:**
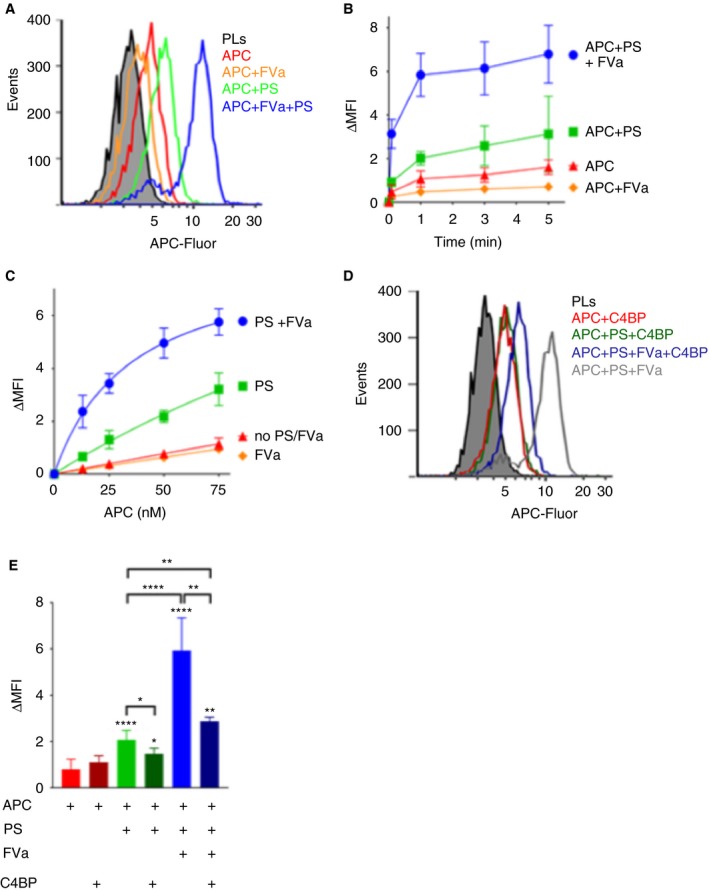
FVa in synergy with protein S enhances the binding of APC to negatively charged phospholipids. A, Representative histograms of binding of 50 nmol/L APC‐Fluor to phospholipid‐coated magnetic beads in the presence and absence of 100 nmol/L protein S and/or 25 nmol/L FVa using flow cytometry. B, The APC association to phospholipid coated magnetic beads was characterized over time. The MFI of the phospholipid‐coated beads was measured at 0, 1, 3, and 5 min after addition of APC. C, APC binding was analyzed at increasing concentrations (0‐75 nmol/L) in the presence and absence of 100 nmol/L protein S and/or 25 nmol/L FVa. Half maximal binding of APC‐Fluor was estimated to be 34.7 nmol/L in the presence of protein S and FVa. D, Representative histograms showing the effects of C4BP upon APC binding to phospholipids. Binding of 50 nmol/L APC‐Fluor to phospholipid‐coated magnetic beads was measured in the presence and absence of 100 nmol/L protein S preincubated with 200 nmol/L β‐chain containing C4BP and/or 25 nmol/L of coagulation factor FVa using flow cytometry. E, Quantification plot of binding of 50 nmol/L APC‐Fluor to phospholipid‐coated magnetic beads in the presence or absence of 100 nmol/L free or C4BP‐bound protein S and/or 25 nmol/L FVa. MFI was measured 1 min after addition of APC. ΔMFI corresponds to the MFI obtained after subtracting the auto fluorescence from the phospholipid coated beads. Data are presented as mean ± SD; n ≥ 3. *P < .05, **P < .01, ****P < .0001 according to Mann‐Whitney tests compared to APC alone, unless otherwise stated

A flow cytometry‐based assay was next developed to quantify APC‐Fluor binding to phospholipid surfaces. For this, phospholipid‐coated magnetic beads were pre‐incubated with/without protein S and FVa, followed by the addition of APC‐Fluor. After 1 minute, any increase in MFI of the phospholipid‐coated beads, corresponding to APC binding, was measured (Figure 2A and E). The interaction of 50 nmol/L APC‐Fluor alone with phospholipids was minimal (Figure 2E). In fact, when titrating APC up to 0.75 μmol/L the binding showed no sign of saturation, suggesting an affinity >0.5 μmol/L (data not shown). This is consistent with previous studies that have reported an affinity ranging from 0.5 to 7.3 μmol/L.^35^, ^36^, ^37^, ^38^ Protein S enhanced the binding of APC‐Fluor to phospholipids moderately (Figure 2A and E), whereas 25 nmol/L FVa alone did not. However, FVa in combination with protein S greatly enhanced the binding capacity (Figure 2A and E), suggesting efficient formation of an APC/protein S/FVa trimolecular complex. APC‐phospholipid association was also measured over time (0‐5 minutes) after addition of APC‐Fluor (Figure 2B). Protein S induced a stable moderate enhancement of APC‐phospholipid binding at all time points. The synergistic enhancement by protein S and FVa was rapid and more pronounced.

These results suggest that the synergistic enhancement of APC binding by protein S, acting together with FVa, arises by increasing the affinity of APC for the membrane surface upon which FVa associates. To further assess this, APC‐Fluor was titrated (0‐75 nmol/L) and its binding to phospholipids analyzed after 1 minute incubation. A dose‐dependent increase in APC binding was observed (Figure 2C). The presence of 25 nmol/L FVa had little or no effect upon APC binding capacity to phospholipid surfaces, whereas APC binding increased modestly in the presence of 100 nmol/L protein S (Figure 2C). However, protein S in combination with FVa appreciably enhanced the APC‐phospholipid association (Figure 2C). Under these conditions, half maximal binding of APC‐Fluor decreased to less than 35 nmol/L, representing a >14‐fold increase in affinity compared to our approximate estimate of APC binding affinity to phospholipids in the absence of protein S and FVa.

### 3.3 Influence of C4BP upon protein S cofactor function

It is well established that protein S bound to C4BP has greatly reduced APC cofactor function.^17^, ^18^, ^19^ We investigated the influence of C4BP‐binding to protein S upon the formation of the APC/protein S/FVa complex. In agreement with functional data,^17^, ^18^, ^19^ C4BP strongly (but not completely) inhibited protein S‐mediated enhancement of APC‐Fluor binding to phospholipids, as well as the synergistic enhancement induced by FVa and protein S together (Figure 2D,E). These findings demonstrate that C4BP binding to protein S disrupts its ability to interact with APC and/or FVa and to assemble into the APC/protein S/FVa complex.

### 3.4 Protein S Gla36 and Asp95 are essential for formation of the APC/protein S/FVa complex formation

Two protein S variants (E36A and D95A) with severely reduced APC cofactor function in plasma and in the inactivation of FVa have been identified.^20^, ^21^ We have previously shown that both variants bind to phospholipids with the same affinity as WT protein S, suggesting that the reason for the decreased APC cofactor function is lack of binding to either APC or FVa.^20^, ^21^ To confirm the results from our previously published data, both variants were studied in thrombin generation and FVa inactivation assays. Using thrombin generation assays in protein S‐depleted plasma, both protein S variants (E36A and D95A), exhibited severely impaired APC cofactor function (Figure S2A). Consistent with this, the ability of both protein S variants to specifically enhance the APC‐mediated cleavage of FVa Arg306 was greatly diminished (Figure S2B; ~8‐9 fold reduction in rate constant of proteolysis according to previous studies^20^, ^21^).

**Figure 3:**
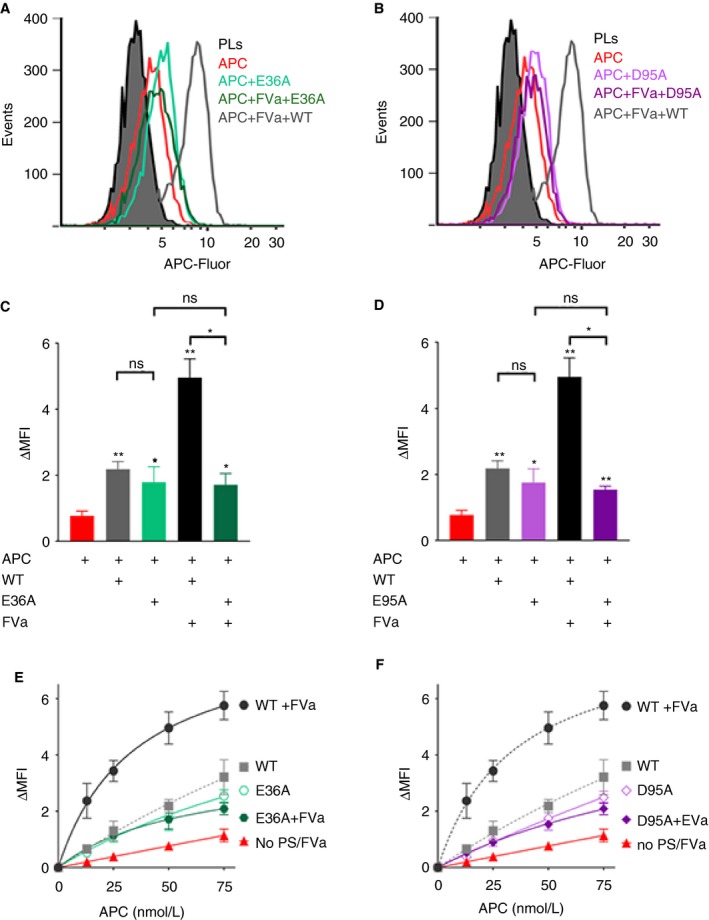
Enhancement of APC binding to phospholipids by protein S variants lacking APC cofactor function. Binding of 50 nmol/L (A‐D) and increasing concentrations (0‐75 nmol/L) (E and F) APC‐Fluor to phospholipid‐coated beads was measured in the presence and absence of 25 nmol/L FVa and 100 nmol/L protein S E36A (A, C, E) or protein S D95A (B, D, F). (A and B) Representative histograms and (C and D) quantification plots of the MFI measured 1 min after addition of APC. ΔMFI corresponds to the MFI obtained after subtracting the auto fluorescence from the phospholipid coated beads. Data are plotted as mean ± SD (n = 3). ns, non‐significant; *P < 0.05, **P < 0.01, according to Mann‐Whitney tests compared to APC alone, unless otherwise stated

The protein S variants were analyzed to study the effect of the mutations upon the assembly of the APC/proteinS/FVa inactivation complex (Figure 3). While both protein S (E36A and D95A) at 100 nmol/L enhanced APC‐Fluor binding to phospholipids in the absence of FVa (similar to WT protein S), no further enhancement was detected in the presence of FVa for either mutant (Figure 3A‐D). This was confirmed by titrating APC‐Fluor (0‐75 nmol/L) in the presence of the protein S (E36A and D95A); see Figure 3E,F. In the absence of FVa, protein S‐mediated enhancement of APC binding to phospholipids by protein S (E36A) or (D95A) was essentially the same as that of WT protein S. Strikingly, no further enhancement of APC binding to phospholipids was detected following addition of FVa to reactions containing either of the variants. These results suggest that in the presence of FVa, protein S residues Gla36 and Asp95 are crucial for the synergistic enhancement of APC binding to membranes and thus essential for formation of the APC/protein S/FVa complex. Moreover, these findings also suggest that Gla36 and Asp95 in protein S may be involved in mediating an interaction either directly with FVa or the APC/protein S/FVa complex, rather than directly with APC.

### 3.5 FV^Nara^ (W1920R) is less efficiently incorporated into the FVa inactivation complex than WT FVa

**Figure 4:**
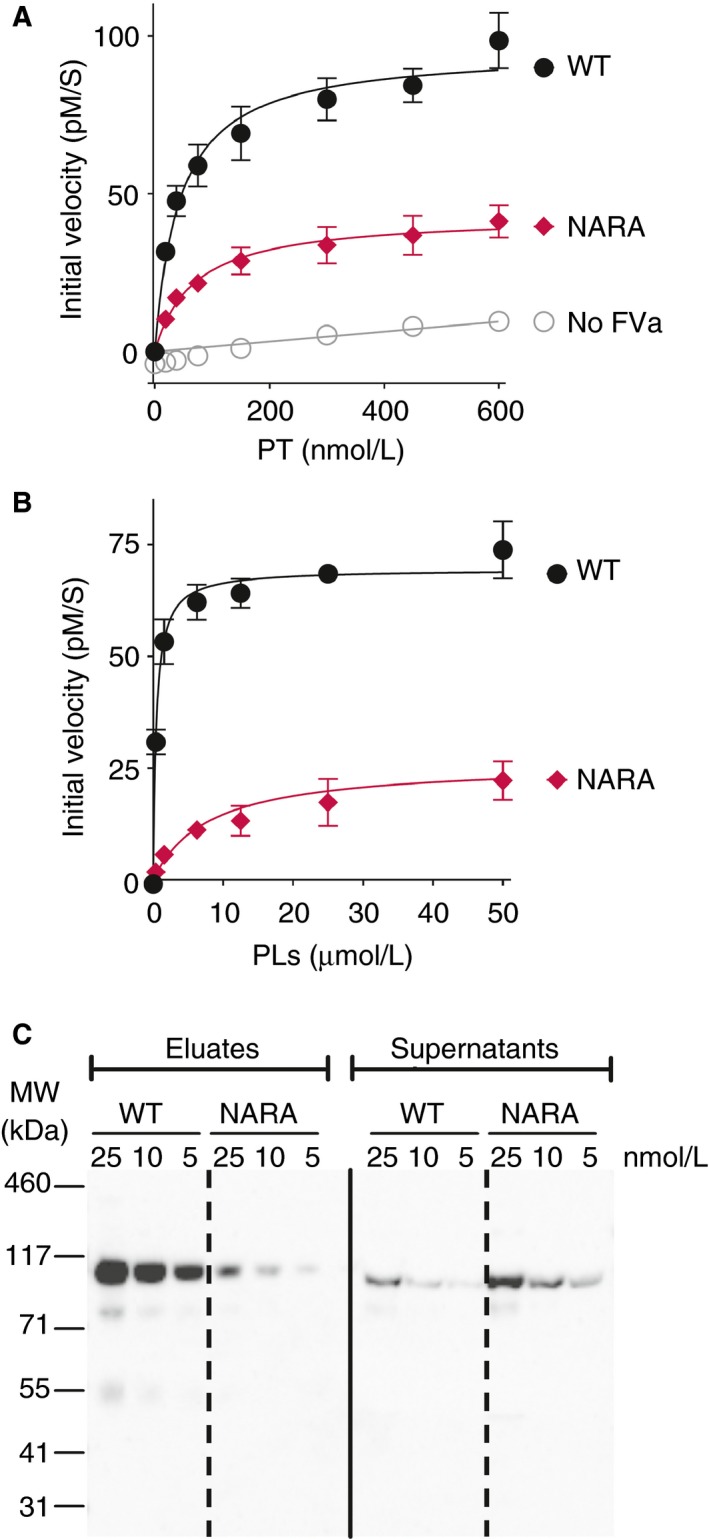
The effect of the FV W1920R mutation in prothrombinase assays and phospholipid binding. (A and B) The ability of FVa^Nara^ to enhance thrombin generation was measured in pure component prothrombinase assays. Thrombin generation was measured in the presence of WT FVa or FVa^Nara^ (8 pmol/L), 5 nmol/L FXa and 50 μmol/L (A) or increasing concentrations of phospholipid vesicles (0‐50 μmol/L) (B). The reaction was initiated by the addition of increasing concentrations (0‐600 nmol/L) (A) or 500 nmol/L of prothrombin (B). C, The effect of the W1920R mutation upon phospholipid binding was evaluated in pull‐down experiment. Phospholipid‐coated magnetic beads were incubated with 5, 10, 25 nmol/L WT FVa or FVa^Nara^ for 25 min. The FVa present in the supernatant (100 μL) and in the bound fraction (30 μL) was analyzed by western blotting (15 μL of each fraction was loaded). Proteins were separated on a 4%‐12% SDS‐PAGE, transferred to nitrocellulose membranes and detected using monoclonal anti‐FV antibodies (AHV‐5146; HTI). The data presented in A‐B are plotted as a mean ± SD (n = 3). The blots shown in C are representative of n = 2. PT, prothrombin; PLs, phospholipids

Prothrombinase assays were performed with FV^Nara^ to assess its ability to function as a cofactor for FXa in the prothrombinase complex. Thrombin generation was initiated using 0‐600 nmol/L prothrombin in the presence of 8 pmol/L FVa or FVa^Nara^ and 5 nmol/L FXa. Conditions were optimized to ensure that the assays were highly FVa‐dependent (ie sensitive to differences in its cofactor function). FVa^Nara^ showed reduced cofactor function (determined as initial velocity) for FXa in the enhancement of thrombin generation (Figure 4A), with a 2.2‐fold reduction in the V_max_ (142 ± 12.2 vs 64.1 ± 14.3 pmol/L/s) for this reaction, and a 2.3‐fold increase in K_m_ (118 ± 57 vs 52 ± 13 nmol/L), when compared to WT FVa. As the cofactor function of FVa in the prothrombinase complex is absolutely phospholipid‐dependent, phospholipids were titrated into prothrombinase assays containing FVa^Nara^. The limiting amounts of FVa or FVa^Nara^ employed made the assays sensitive to changes in FVa affinity for phospholipids. While a decrease (~40%) was detected in the V_max_ of thrombin generation for FVa^Nara^ compared to WT FVa (Figure 4B), a 16‐fold increased K_d_ for phospholipids (8.5 ± 0.5 μmol/L vs 0.5 ± 0.1 μmol/L) was also observed.

The ability of FVa^Nara^ to bind to phospholipids was also evaluated using pull‐down experiments (Figure 4C). More WT FVa bound to phospholipid‐coated beads than FVa^Nara^, which was supported by the increased amounts of unbound FVa^Nara^ in the supernatant compared to WT FVa. Together these findings confirm that FVa^Nara^ exhibits reduced affinity for phospholipid surfaces.

**Figure 5:**
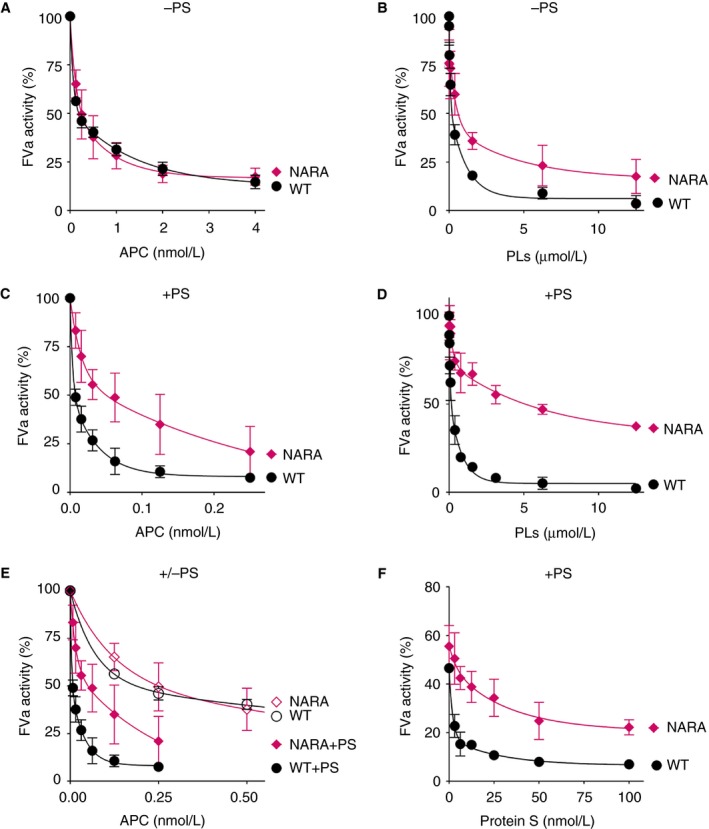
Characterization of APC‐mediated inactivation of FVa^Nara^. Inactivation of FVa^Nara^ by APC was assessed in FVa inactivation assays. FVa (0.8 nmol/L) was inactivated in the absence (A,B) or presence (C‐F) of protein S at various concentrations of APC, protein S, and phospholipid vesicles. A,B, FVa inactivation was measured in the absence of protein S at increasing concentrations of APC (0‐4 nmol/L) in the presence of 25 μmol/L phospholipid vesicles (A) or in the presence of increasing concentrations of phospholipid membranes (0‐12.5 μmol/L) and 5 nmol/L APC (B). C,D, FVa inactivation was also investigated in similar assays in the presence of protein S. Here FVa inactivation was performed at increasing concentrations of APC (0‐0.25 nmol/L) in the presence of 25 μmol/L phospholipid vesicles (C) or in the presence of increasing concentrations of phospholipid membranes (0‐12.5 μmol/L) and 0.25 nmol/L APC (D). E, Data from A and C are merged to better visualize reduced protein S enhancement of FV^Nara^ in assays run in the presence of high phospholipid concentration (25 μmol/L). F, FVa inactivation by 0.25 nmol/L APC was also quantified in the presence of increasing concentrations of protein S (0‐100 nmol/L) in the presence of phospholipid vesicles (25 μmol/L). All inactivation reactions were allowed to proceed for 10 min. Remaining FVa activity was quantified using prothrombinase assays as described in the methods section. 100% corresponds to FVa activity in the absence of APC in all graphs. The data is presented as mean ± SD (n = 3). PS, protein S; PLs, phospholipids

Intriguingly, although FVa^Nara^ has reduced cofactor function for FXa in the prothrombinase complex, the W1920R substitution exerts a prothrombotic phenotype. To reconcile this, the ability of FV^Nara^ to be proteolytically cleaved by APC was studied in FVa inactivation assays (Figure 5). Initially, WT FVa and FVa^Nara^ were inactivated in the presence of 0‐4 nmol/L APC, in the absence of protein S. Under these conditions FVa^Nara^ was inactivated by APC in a manner indistinguishable from that of WT FVa (Figure 5A). To determine the effect of the reduced phospholipid binding of FV^Nara^ upon the inactivation reaction, we inactivated WT FVa and FVa^Nara^ with 5 nmol/L APC at increasing concentrations of phospholipids (Figure 5B). As expected, the inactivation was enhanced by increasing phospholipid concentration. Furthermore, the results confirmed the reduced affinity of FVa^Nara^ for phospholipid membranes compared to WT FVa (Figure 5B). However, at high phospholipid concentrations this difference was minimal, explaining why FVa^Nara^ behaved as WT FVa in Figure 5A. Next, we analyzed the inactivation of WT FVa and FVa^Nara^ by increasing concentrations of APC (0‐0.25 nmol/L) in the presence of protein S. In contrast to the results obtained in the absence of protein S, we observed a clear reduction of FVa^Nara^ inactivation compared to WT FVa (Figure 5C). In these assays ~6 pmol/L APC reduced the WT FVa activity by ~50%, compared to >50 pmol/L for similar reduction of FVa^Nara^ activity (Figure 5C). These results suggest that the resistance of FVa^Nara^ to APC proteolysis is attributable to the decreased enhancement by protein S. Because the enhancement by protein S is completely phospholipid dependent, we studied the inactivation of both FVa variants by APC (0.25 nmol/L) at increasing phospholipid concentration also in the presence of 100 nmol/L protein S (Figure 5D). FVa^Nara^ inactivation was once again reduced compared to WT FVa. However, in the presence of protein S the reduction in inactivation was much more pronounced. We therefore plotted the data obtained from the APC titrations in the presence and absence of protein S together (Figure 5E). These experiments were performed at high phospholipid concentrations (25 μmol/L) and can thus be directly compared. When comparing the inactivation of WT FVa and FVa^Nara^, it appears as if the protein S enhancement of FVa^Nara^ inactivation is reduced compared to that of WT FVa. This was confirmed in assays in which we investigated WT FVa and FVa^Nara^ inactivation by 0.25 nmol/L APC in the presence of high concentrations of phospholipids (25 μmol/L) and increasing concentrations of protein S (Figure 5F). In these assays, 3 nmol/L protein S markedly augmented WT FVa inactivation. To achieve comparable levels of FVa^Nara^ inactivation, >50 nmol/L protein S was required. These results suggest that the reduced inactivation of FVa^Nara^ observed in Figures 5C‐F is unlikely to be related to decreased phospholipid binding alone, but rather to a combination of decreased affinity of FVa^Nara^ toward both the phospholipid membranes and protein S. Of note, Nogami and colleagues showed lack of cleavage of FVa^Nara^ at Arg306, which is completely dependent upon phospholipids and enhancement by protein S.^12^

Taken together, these results show that the W1920R mutation causes a decrease in binding to phospholipids but, more importantly, specifically show reduced enhancement by protein S of APC‐mediated inactivation of FVa^Nara^ compared to WT FVa.

**Figure 6:**
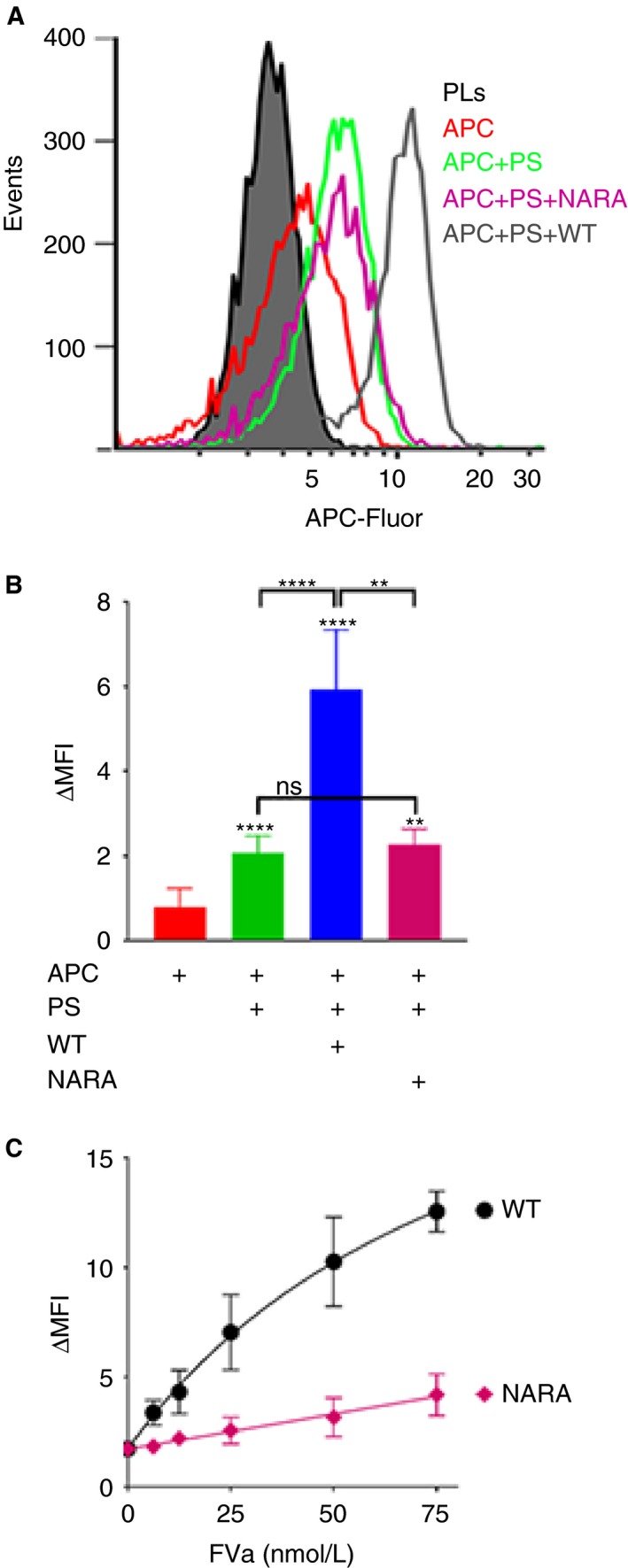
Ability of FVa^Nara^ to enhance the formation of the APC/protein S/FVa trimolecular complex. Binding of 50 nmol/L APC‐Fluor to phospholipid‐coated magnetic beads was characterized in the presence and absence of 100 nmol/L protein S and/or 25 nmol/L WT FVa or FVa^Nara^ (A and B) or with increasing concentration of FVa WT or FVa^Nara^ (0‐75 nmol/L) in the presence of 100 nmol/L protein S (C) using flow cytometry. A, Representative histograms and (B and C) quantification plots of the MFI measured 1 min after addition of APC. ΔMFI corresponds to the MFI obtained after subtracting the auto fluorescence from the phospholipid coated beads. The data is presented as mean ± SD (n** **= 3). ns, non‐significant; **P < 0.01, ****P < 0.0001 according to Mann‐Whitney tests compared to APC alone, unless otherwise stated

To examine FV^Nara^ incorporation into the inactivation complex, binding of 50 nmol/L APC‐Fluor to phospholipid‐coated beads was measured in the presence of 100 nmol/L protein S, 25 nmol/L FVa/Va^Nara^ (Figure 6A,B). Compared to WT FVa, FVa^Nara^ showed severely reduced ability to synergistically enhance, with protein S, the association of APC to phospholipids. When FVa^Nara^ was titrated (0‐75 nmol/L) in the presence of 100 nmol/L protein S and 50 nmol/L APC, 50 nmol/L FVa^Nara^ enhanced APC‐phospholipid binding less effectively than 6 nmol/L WT FVa (Figure 6C). Together with the FVa inactivation assay data, these results demonstrate that substitution of Trp1920 in FV impairs protein S‐dependent enhancement of APC, and in turn, severely compromises formation of the APC/protein S/FVa inactivation complex on negatively charged phospholipid membranes.

## 4 DISCUSSION

The APC/protein S/FVa complex is short‐lived as it dissociates once FVa is proteolyzed, making direct detection of this complex difficult. Moreover, its formation is phospholipid‐dependent, which limits the methods available for its analysis. A flow cytometry‐based method has been developed here to study the association of fluorescently labelled/inactive APC with phospholipid surfaces, and therefore also the formation of the APC/protein S/FVa inactivation complex. The limited binding of APC to the phospholipid membranes in the absence of its substrate (FVa) and cofactor (protein S), is in agreement with its comparatively low (μmol/L range) affinity for phospholipids, particularly when compared to other Gla domain containing proteins.^35^, ^36^, ^37^, ^38^ As previously suggested, protein S increased association of APC to phospholipid surfaces (Figures 1 and 2A‐C and E).^24^ However, the enhancing effect of protein S was rather modest, and by itself is insufficient to account for the full functional enhancement that protein S exerts upon APC in functional assays. Interestingly, APC association with phospholipid surfaces was greatly enhanced in the presence of both protein S and FVa (Figures 1 and 2A‐C and E). This is in agreement with Smirnov et al., who reported a similar synergistic enhancement of APC binding to phospholipids by protein S and FVa in a manner that was dependent on phosphatidylethanolamine.^38^ FVa, in the absence of protein S, did not increase the APC/phospholipid association, showing that protein S is absolutely required for efficient enhancement. In agreement with results of functional assays, the complex is formed rapidly.^39^, ^40^ Our findings therefore point to the rapid formation of a high‐affinity, phospholipid‐dependent, APC/protein S/FVa complex. The apparent affinity of APC in this complex for phospholipids (~35 nmol/L) is markedly higher that the affinity of APC alone for phospholipids, which from our titrations appears to be >0.5 μmol/L, agreeing with previous studies which range from 0.5 to 7.3 μmol/L.^35^, ^36^, ^37^, ^38^ Accordingly, this represents a >14‐fold enhancement of affinity of APC binding to phospholipids in the presence of both protein S and FVa. While protein S is important for the enhancement of binding, it is clear that FVa also plays a critical role in the recruitment of APC to the phospholipid membrane and, in this way, plays a pivotal role in its own inactivation. In addition to FVa, APC also inactivates FVIIIa. Whether similar mechanisms are involved in the APC‐mediated inactivation of FVIIIa remains to be investigated.

Free protein S (rather than C4BP‐bound protein S) is considered to be the major anticoagulant pool of protein S. Consistent with this, we demonstrate that C4BP‐bound protein S has reduced ability to assemble into the tri‐molecular complex with APC and FVa (Figure 2D,E). The protein S SHBG‐like domain contains the binding site for C4BP, but has also been proposed to harbor an interaction site for FVa.^41^, ^42^, ^43^ It is thus plausible that C4BP blocks an important protein S/FVa interaction^44^ and thereby inhibits the formation of the APC/protein S/FVa inactivation complex. It should be considered though that C4BP is a large (~500 kDa) multimeric glycoprotein that may exert its effects through steric hindrance rather that direct competition for the same interaction site.

**Figure 7:**
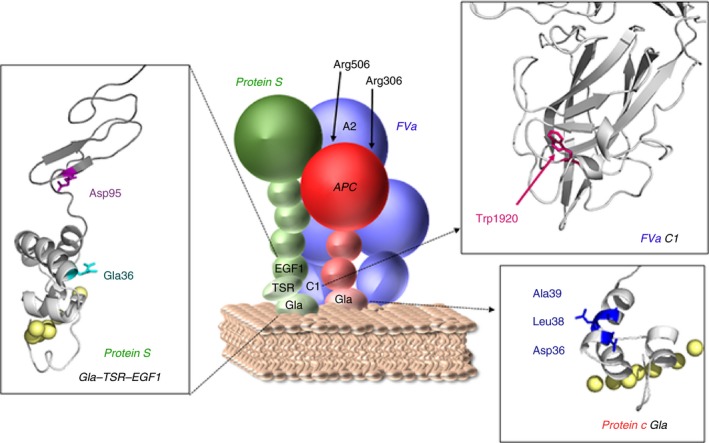
Schematic model of the proposed inactivation complex, involving protein S Gla36, Asp95, protein C Asp36, Leu38, Ala39, and FV Trp1920. Our results suggest that protein S residues Gla36, Asp95, and FV Trp1920 are crucial for formation of the inactivation complex among APC, protein S, and FVa. A previously published paper showed that protein C Asp36, Leu38, and Ala39 are essential for APC to be enhanced by protein S and were therefore suggested to be involved in a direct interaction with protein S.^22^ A cartoon of the complex is shown where APC is represented in red, protein S in green, and FVa in blue. Models of the protein S Gla‐TSR‐EGF1 and FVa C1 are shown in inserts. The models are in gray with protein S Gla36, Asp95, and FV Trp1920 shown in red, purple and pink, respectively. The predicted coordinated calcium ions in the protein S Gla domain are shown as yellow spheres. The protein S Gla‐TSR‐EGF1, APC, and FVa models are taken from Villoutreix et al.,^49^ Mather et al.,^50^ and from Lee et al.,^51^ respectively

Protein S (E36A and D95A) both exhibit greatly impaired APC cofactor function (Figure S2).^20^, ^21^ These variants were investigated to characterize their influence upon APC binding in the formation of the inactivation complex (Figure 3). Both protein S variants are known to bind to negatively charged phospholipid membranes with the same affinity as WT protein S, suggesting that these residues are likely involved in intermolecular interactions with FVa (and/or APC), rather than being involved in membrane binding.^20^, ^21^ Both residues are in close proximity to protein S residues 37‐50, which have been suggested to form part of an interaction site for FVa.^45^ Binding studies of protein S variants (E36A and D95A) revealed that these mutations are unlikely to be involved in any direct interaction with APC, as the modest enhancement of APC binding to phospholipids by protein S was essentially unaffected by these substitutions (Figure 3C,D). However, the mutations abolished the synergistic enhancement of APC binding to phospholipids mediated by protein S and FVa acting together. These results suggest that protein S Gla36 and Asp95 are potentially involved in a direct FVa interaction site, possibly related to that suggested by Heeb and colleagues.^45^ These protein S residues are located in the Gla domain and EGF1 domain of protein S, suggesting that the corresponding interaction site in FVa may involve amino acids that are in proximity to the membrane‐binding region of FVa (Figure 7).

Nogami et al. recently identified the homozygous FV^Nara^ mutation (FV W1920R) in a boy with recurrent DVT, whose phenotype was attributed to severe APC resistance of the mutant FV.^12^ The authors suggested that APC resistance may be caused by disruption of an APC interaction site. However, Trp1920 is located in the conserved C1 domain (Figure 7), in proximity to the proposed phospholipid binding sites.^46^, ^47^ Results from both prothrombinase and FVa inactivation assays, as well as pull‐down phospholipid binding assays, all reflect the impaired phospholipid binding of FVa^Nara^ (Figures 4 and 5), which is in contrast to the previously published data.^12^ This is likely due to differences in experimental approach: attention is drawn to careful optimization of assays in the present study such that they were sensitive to differences in phospholipid binding affinity. In our assays, phospholipids were titrated in the presence of low (8 pmol/L) FVa, whereas Nogami and colleagues used 2 nmol/L FVa (250‐fold higher concentration). High FVa concentrations likely diminish the functional consequences associated with reduced phospholipid‐binding of FV^Nara^. Intriguingly, although the reduced phospholipid binding of FVa^Nara^ reduces its prothrombinase function, the W1920R mutation phenotype appears to be procoagulant overall due to a profound reduction in APC‐mediated inactivation of FVa^Nara^ and the FV^Nara^ dependent inactivation of FVIIIa.^12^ However, we show that the resistance to APC‐mediated FVa^Nara^ proteolysis was only observed in the presence of limited concentrations of negatively charged phospholipid membranes and/or protein S (Figure 5B‐F). In the absence of protein S and at saturating amounts of phospholipids, the inactivation of FVa^Nara^ was essentially normal (Figure 5A,B). Protein S and negatively charged phospholipid membranes are important for enhanced inactivation of FVa at Arg306, which previously has been shown to be absent in the inactivation of FVa^Nara^.^12^ While the current findings show that the FV W1920R mutation causes decreased affinity for phospholipids, this is only revealed at low/limiting phospholipid concentrations. Unlike the procoagulant deficit in FVa^Nara^, the reduction in synergistic enhancement of APC binding to phospholipids with protein S is clear even at high phospholipid concentrations (Figures 5 and 6). Our results, therefore, suggest that despite reduced phospholipid binding affinity, which would also influence its procoagulant effects, the primary phenotypic effect of FV^Nara^ is its decreased APC cofactor function, due to reduced affinity for protein S. We therefore demonstrate that the APC resistance associated with FV^Nara^ is a consequence of its reduced ability to be incorporated into the trimolecular complex with APC and protein S.

The present study provides insight into the formation of an APC/protein S/FVa inactivation complex. It is proposed that functional interaction sites between these molecules are located proximal to the membrane binding domains of the proteins, in protein S Gla‐EGF1 and FV C1 domain and APC Gla domain regions (Figure 7).^22^ This may in turn facilitate the movement required to enable the APC serine protease domain to access the three cleavage sites, and associated binding sites,^48^ in the FVa A2 domain. It is now evident that all three proteins, enzyme, cofactor, and substrate, have a critical role in the formation of the inactivation complex and that this is essential for the function of the APC anticoagulant pathway.

## CONFLICT OF INTEREST

The authors declare no competing financial interests.

## AUTHOR CONTRIBUTIONS

M.G. and J.A. designed the research, analyzed the results, and wrote the paper. M.G., I.I.S‐C., S.S., and A.T‐O. performed the experiments. I.I.S‐C., S.S., J.T.B.C., and D.A.L. designed the research and revised the manuscript.
