# Supplementary material for: The roles of factor Va and protein S in formation of the activated protein C/protein S/factor Va inactivation complex
Source: J Thromb Haemost. 2019 Aug 9;17(12):2056–68. doi: 10.1111/jth.14594 (PMC6916587; doi:10.1111/jth.14594)
Supplement: Supplementary file 1 [file JTH-17-2056-s001.pdf]

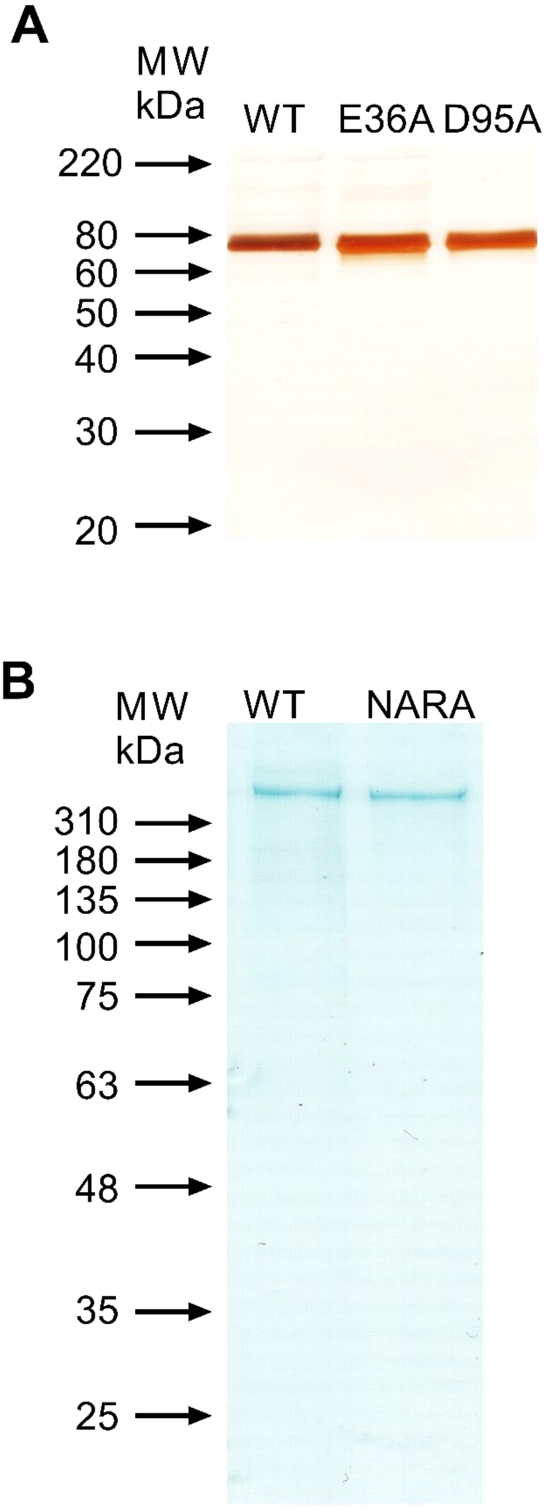

Supplementary Figure 1. SDS-PAGE analysis of purified recombinant protein S and FV. (A) 1µg of purified WT protein S and protein S variants E36A, D95A were separated on 10% SDS-PAGE under non-reducing conditions. Proteins were visualised using silver staining. **(B)** 1.5µg of purified, recombinant full-length WT FV and FV NARA were analysed on 10% SDS-PAGE under non-reducing conditions. Proteins were detected using InstantBlue® stain (Expedeon). MW, molecular weight.

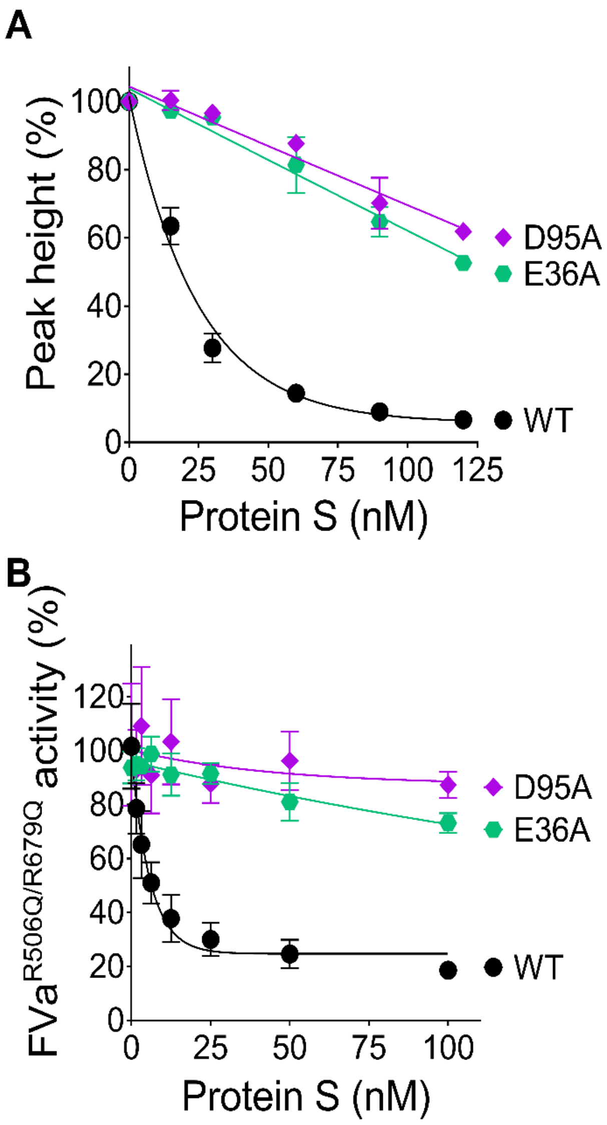

Supplementary Figure 2. Characterization of APC cofactor function of protein S variants by thrombin generation and FVa inactivation assays. (A) Thrombin generation was measured using CAT after initiation by 1pM TF in protein S-depleted plasma supplemented with phospholipids, 9nM APC, and WT protein S, protein S E36A or protein S D95A. **(B)** The ability of protein S to enhance APC-mediated cleavage of FVa at Arg306 was evaluated in FVa inactivation assays. FV R506Q/R679Q (0.8nM) was inactivated by 0.25nM APC with increasing concentrations of protein S (0-100nM) for 10 minutes. Remaining FVa activity was measured in prothrombinase assays. Results are presented as a mean ± SD (n=3). 100% corresponds to the peak height (**A**) or FVa activity (**B**) in the absence of APC in all graphs.
